# The Mediterranean Lifestyle as a Non-Pharmacological and Natural Antioxidant for Healthy Aging

**DOI:** 10.3390/antiox4040719

**Published:** 2015-11-12

**Authors:** Kyriazoula Chatzianagnostou, Serena Del Turco, Alessandro Pingitore, Laura Sabatino, Cristina Vassalle

**Affiliations:** Fondazione CNR-Regione Toscana G. Monasterio and Institute of Clinical Physiology-CNR, Pisa 56124, Italy; E-Mails: zulagr71@gmail.com (K.C.); serena@ifc.cnr.it (S.D.T.); pingi@ifc.cnr.it (A.P.); laura.sabatino@ifc.cnr.it (L.S.)

**Keywords:** Mediterranean diet, oxidative stress, antioxidants, aging, chronic degenerative diseases

## Abstract

Oxidative stress has been suggested to affect age-associated physiological dysfunction. Therefore, it is speculated that antioxidant supplements could have a potential role in preventing age-related diseases and death. Among different dietary habits, the highly antioxidant Mediterranean dietary pattern, which includes high vegetable and fruit intake, consumption of legumes, cereals, and fish, low intake of meat and dairy derivatives, moderate red wine consumption, and use of extra-virgin olive oil, is characterized by other aspects than food, such as conviviality, sensory stimulation, socialization, biodiversity, and seasonality that can reinforce the Mediterranean diet’s (MeD) beneficial effects on wellbeing, quality of life, and healthy aging. The present review aims to discuss available data on the relationship between oxidative stress and aging, biomarkers of oxidative stress status, protective effects of the MeD, and the adoption of the Mediterranean lifestyle as a non-pharmacological and natural tool to cope with oxidative stress damage for a longer life span, and—even more important—healthy aging beyond the biological, psychological, and social challenges that old age entails.

## 1. Introduction

Aging is an unavoidable, complex, and multifactorial event leading to progressive loss of function, disability, and death. Although the biological basis of aging is unknown, oxidative stress is a central determinant in aging theories [1]. Thus, the possibility to combat oxidative stress with antioxidant strategies—diet, exogenous antioxidant supplementation, or lifestyle changes—appears as an exciting tool to improve wellbeing and health for healthy aging.

Among different dietary habits, the highly antioxidant Mediterranean diet (MeD) is characterized by a high vegetable and fruit intake, consumption of legumes, cereals, and fish, low intake of meat and dairy derivatives, moderate red wine consumption, and use of extra-virgin olive oil [2]. Moreover, UNESCO in 2010 has officially defined MeD as a cultural heritage of humanity, exalting conviviality, sensory stimulation, socialization, biodiversity, and seasonality, aspects that can reinforce the MeD’s beneficial effects on wellbeing, quality of life, and health [3,4]. The present review aims to discuss available data on the relationship between oxidative stress and aging, biomarkers to assess the oxidative stress status, protective effects of the MeD, and the adoption of the Mediterranean lifestyle as a non-pharmacological and natural tool to cope with oxidative stress damage and prevent aging as well as major degenerative and chronic diseases (cancer, neurodegenerative and cardiovascular (CV) disease).

## 2. Oxidative Stress and Aging

Reactive oxygen species are normally produced in small quantities during physiological processes consequent to energy production and as the result of molecular oxygen reduction to water. However, excessive free radical production or impaired capacity of endogenous antioxidants to detoxify or repair the oxidative injury leads to oxidative stress [5,6]. This status increases the damaging potential level of ROS directed against the main macromolecules (carbohydrates, lipids, nucleic acids, and proteins) and cellular components (membranes, organelles, the nucleus, and free enzymes) [6]. These oxidative stress changes have been considered important factors in the physiopathology of aging and the major chronic diseases.

High ROS levels react with lipids to induce the release of lipid peroxides that decompose to form numerous products including malondialdehyde (MDA) and isoprostanes, measurable in biological samples [7,8]. MDA is one of the end-products of lipid peroxidation and is a well-established marker of oxidative damage in degenerative chronic diseases. An excess of MDA can produce adducts with free amino groups of proteins, lipids, and nucleic acids, altering their biological properties. Elevated levels of 8-iso prostaglandin F2α (8-isoprostane), derived from free-radical peroxidation of arachidonic acid, have been reported in many age-related diseases [6]. Moreover, isoprostanes may also act as pathophysiologic mediators of oxidant injury, causing vasoconstriction of blood vessels and influencing aggregation of platelets [8].

As a marker of oxidative damage to proteins, carbonyls have been shown to accumulate during aging, chronic inflammation, and many age-related diseases [9]. Protein carbonyls may be generated by the oxidation of several amino acid side chains or by glycation/glycoxidation of Lys amino groups, forming advanced glycation end products, major contributors of initiation, and development of many age-related events [10,11].

There is recent evidence of a correlation between ROS levels and the rate of telomere shortening. Telomeres, repetitive DNA sequences of (TTAGGG)*_n_* located at the ends of eukaryotic chromosomes, are essential to the stability of chromosome and cell replication. The progressive shortening of telomeres leads to senescence, apoptotic cell death, or the oncogenic transformation of somatic cells in various tissues [12].

Among the biological structures progressively affected by aging, the endothelium is one of the most important and alterations of enzymatic function, such as endothelial nitric oxide synthase (NOS) activity, interfere with endothelial-dependent dilation, and, as a result, vascular homeostasis [13]. The impairment of endothelial function is also characterized by chronic low-grade inflammation that by itself promotes cellular oxidative stress. ROS interact with different redox-sensitive transcriptional factors, such as activator protein 1 (AP-1) and nuclear transcription factor-kappa B (NF-κB), involved in gene expression of proteins that regulate cell proliferation and inflammation (adhesion molecules, cytokines), promoting endothelial apoptosis [14]. When damaged, the endothelium typically releases microparticles (MPs), cell-derived membrane vesicles that carry surface and cytoplasmic proteins of the parental cells, and act as biological messengers. Endothelial MPs, in turn, promote cellular senescence through an increased ROS production, thus emerging as an attractive biomarker of vascular damage and endothelial dysfunction [15,16]. 

**Figure 1:**
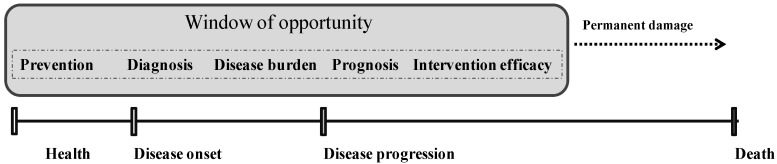
Use and significance of biomarkers during the onset and development of disease. Biomarkers can be used for risk estimation, diagnosis, and determination of severity and pharmacological treatment/lifestyle intervention efficacy.

All these oxidative stress biomarkers can be measured in biological samples before onset and during disease progression, making them potentially helpful tools for assessing risk, diagnosing, determining disease severity and prognosis, and responding to treatments/interventions before permanent damage (Figure 1) [6]. In particular, biomarkers of risk and prognostic markers may identify subjects at high risk for disease onset (in those without disease) or disease progression (in those with existing disease). At diagnosis, a biomarker would distinguish between subjects with and without disease with good sensitivity and specificity. A burden of disease biomarker would help to stratify disease severity in patients with the disease. A biomarker to test the efficacy of intervention can be used to assess the short- or long-term changes associated with pharmacological treatments/lifestyle interventions. Many oxidative stress biomarkers have been found promising in many aspects, although further insights and validation approaches would greatly enhance our knowledge of their role in aging and disease and help to extend their application and significance in the clinical practice [6].

Another important mechanism by which oxidative stress and longevity are modulated seems to be related to sirtuin (SIRT) activation [17]. In particular, SIRT1, belonging to the sirtuin family, is emerging as a key factor in aging and oxidative stress status, along with NAD-dependent histone deacetylase activity and regulation of multiple protein transcriptions [17]. Moreover, the aging process is associated with a progressive reduction in SIRT1 activity, while increased expression or activation of SIRT1 is related to prolonged life-span in minor animal models [17]. Increased SIRT1 expression has been related to antioxidant upregulation, downregulation of pro-apoptotic factors through the involvement of Forkhead box O transcription factors (Fox01-06, with capacity to detoxify ROS and repair DNA damage), and oxidative stress reduction. Moreover, SIRT1 negatively regulates p66Shc, which increases intracellular ROS levels through oxidoreductase activity, and NF-κB, whose DNA-binding capacity is decreased by deacetylation of its RelA/p subunit by SIRT1, which affects the expression of genes involved in inflammatory responses and cellular proliferation [17,18]. By targeting all these proteins, SIRT1 activity is related to a number of critical signaling pathways, including metabolic control, DNA repair apoptosis, cell survival, development, inflammation, mitochondrial function, hormones secretion, neurogenesis, mitochondrial biogenesis, senescence, and tolerance to oxidative stress [17,19].

**Figure 2:**
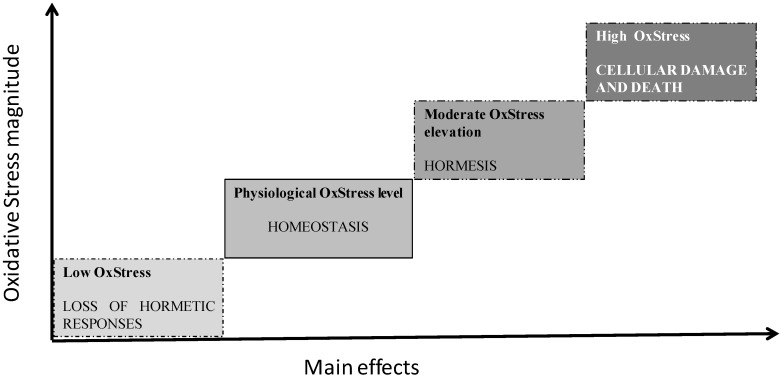
Oxidative stress magnitude and corresponding main effects.

Conversely, it is important to note that there is a positive counterpart to the role of some ROS: they are critical functional coordinators of cellular activities and regulators of signaling pathways [20]. Therefore, moderate oxidative stress is not necessarily an adverse condition, since its consequences may be beneficial for many physiological reactions in cells [20,21]. Specifically, moderate ROS elevation may be protective against ROS-induced oxidative stress in the so-called hormetic response, a molecular mechanism to stress adaptation [21]. According to this hypothesis, some repeated noxious stimuli in the environment could upregulate antioxidants and maintain a more reducing environment in response to oxidative stress bursts [2]. Thus, ROS can be beneficial if present in moderate quantity, and dangerous if in excess. By contrast, lowering the levels of oxidative stress, for example by excessive antioxidant supplementation intake, may be potentially harmful because this impairs the hormetic response [2]. Thus, the balance between ROS and antioxidants may be considered optimal at physiological/moderate levels, but excessive oxidants, as well too low oxidative stress, are both potentially dangerous (Figure 2).

## 3. Antioxidant Supplementation and Healthy Aging

As antioxidants represent the main barrier to oxidative stress injury and are considered to slow down the aging process, the use of multivitamin/mineral supplements has grown exponentially in recent years. Although many experimental data suggest that supplementation with vitamin C and E induces an increase in life span, other studies did not find any benefit, and others even suggested a reduction in life span in response to vitamin E supplementation [22,23]. Interestingly, recent experimental data showed that 500 mg vitamin E/kg diet in LDL receptor-deficient (LDRL(−/−)) mice was not effective at reducing mortality and atherosclerosis when the diet contained high or medium levels of fat and cholesterol. However, a relatively low dose and long-term vitamin E supplementation from an early age was effective in reducing mortality and atherosclerotic lesions in genetically prone LDLR(−/−) mice fed a low fat/cholesterol diet, thus demonstrating that antioxidant supplementation is not sufficient unless integrated with a complete healthy diet plan [24].

In humans, available data on beneficial effects of antioxidant supplementation are even more controversial, as results did not clearly demonstrate significant beneficial effects of antioxidant supplementation but rather, in some cases, increased mortality [25]. Recent data from the Leisure World Cohort Study (8640 women and 4983 men; median age at enrolment: 74 years; follow-up 1981–2013), showed that neither dietary nor supplemental intake of vitamins A, C, and E was significantly associated with mortality [26]. Supplementation with vitamin E, analyzed in a meta-analysis, appears to have no effect on all-cause mortality at doses up to 5500 IU/day [27]. Also, in the Framingham Study, supplemental vitamin E did not protect against risk for CV disease and mortality in 4270 CV disease patients [28]. Similarly inconclusive results have been obtained in literature reviews focused on cognitive function, where supplementation appears efficacious against cognitive decline only in elderly subjects with low status of vitamins (folate < 12 nmol/L or vitamin E intake < 6.1 mg/day), although no definitive role emerges for vitamin B12 [29]. Other data suggested that two-year folic acid and vitamin B12 supplementation did not affect cognitive performance in elderly people with elevated homocysteine levels (2919 elderly participants, ≥65 years) [30]. Conversely, high folate or folic acid supplementation was detrimental to cognition in older people with low vitamin B12 levels in another study (1354 elderly subjects) [31]. 

Clearly, discrepancies among results may be due to species-specific differences, and, in the case of human subjects, to the characteristics of the patients enrolled [32]. However, the possible harmful effects of exogenous antioxidant supplementation may be related to the high vitamin dosage, the duration of supplementation (the effects of long-term exogenous antioxidant supplementation on health are unknown) and whether vitamins are used alone or as part of a combined antioxidant cocktail [32]. Moreover, other points need to be considered, such as the possible interaction with other exogenous antioxidants or foods retaining antioxidant capacity. Indeed, some results suggested that antioxidant supplementation may induce adverse pro-oxidant effects [33]. Thus, since some antioxidant compounds could express pro-oxidant potentials, depending on their levels and on the microenvironment, accurate determination of an individual’s oxidative stress status is necessary before deciding about possible antioxidant supplementation [2,32,33]. It is also important to consider that oxidative stimuli can be beneficial according to the hormesis theory, which describes a biological beneficial response after exposure to low doses of an agent that is dangerous when given at high doses. 

In addition, dietary recommendations targeted to ensure sufficient intake to prevent deficiency do not necessarily lead to an appropriate reduction in the risk of chronic disease. 

Instead, natural intake of antioxidant molecules through diet, such as omega-3-fatty acids and pholyphenols contained in fish or fruit and vegetables, respectively, and present in the typical foods of the Mediterranean dietary pattern, exerts beneficial effects on aging, CV disease, cancer, and cognitive loss [34,35,36,37].

## 4. Mediterranean Diet and Healthy Aging

The traditional Mediterranean lifestyle characterized for centuries the habits and culture of the population living around the Mediterranean Sea, who survived by working hard and applying frugality in food consumption. This lifestyle has been officially defined as a cultural heritage of humanity, having other aspects than food—such as conviviality, sensory stimulation, socialization, biodiversity, and seasonality—that can support the MeD’s beneficial effects on wellbeing, quality of life, and healthy aging [3,4]. Adherence to the traditional MeD is associated with low mortality (higher longevity) and reduced risk of developing chronic diseases, including cancer, metabolic syndrome, depression, and CV and neurodegenerative diseases [37,38,39].

In particular, recent results showed that strict adherence to MeD, non-smoking, and physical activity in an Italian population during a 20-year follow-up study (1693 subjects aged 40–74) were significantly associated with a reduced risk of all-cause mortality, and this reduction was even stronger when the healthy lifestyle behaviors were combined [40]. In the 1290 participants of the Aragon Workers Health Study cohort, closer adherence to the Mediterranean dietary pattern was associated with an improved lipid profile when compared with a Western dietary pattern [41].

MeD adherence has been found to be beneficial with regards to biomarkers of CV risk [42,43,44]. In particular, the PREDIMED (PREvención con DIeta MEDiterránea) results, which reported the long-term effects of the MeD on CV events in patients at high CV risk (with diabetes or more traditional CV risk factors), showed the favorable effects of the MeD + extra-virgin olive oil and MeD + nuts on blood pressure, lipid profiles, lipoprotein particles, inflammation, oxidative stress, and carotid atherosclerosis, as well as on the expression of pro-atherogenic genes involved in CV injury and thrombosis [42,43]. Interestingly, both MeDs showed interactions with several genetic variants (cyclooxygenase-2, interleukin-6 (IL-6), apolipoprotein A2, cholesterol ester transfer protein plasma, and transcription factor 7-like 2 gene polymorphisms) in nutrigenomic studies [42,43]. Moreover, medium or high adherence to MeD was found to decrease diabetes risk by 49% and 62%, respectively, compared with low MeD adherence [43]. Higher MeD adherence was more beneficial for subjects with higher waist circumference (>94 for men, >80 for women) [43]. Wholegrain cereals, fruits, and legumes had the highest positive predictive power [43]. The anti-diabetic effect of MeD was associated with lower levels of oxidative and inflammatory biomarkers such as tumor necrosis factor-α, as well as homocysteine and total antioxidant capacity [43]. A significant reduction of type 2 diabetes has been found to be associated with greater consumption of healthy dietary patterns, including the MeD, the DASH (Dietary Approach to Stop Hypertension) diet, and the AHEI (Alternative Healthy Eating Index) [45].

In a subgroup of the PREDIMED (PREvención con DIeta MEDiterránea) trial, patients at high CV risk but without diabetes (3541 patients aged 55 to 80 years), were randomly assigned to a low-fat diet or two types of MeD, supplemented either with extra-virgin olive oil (1 L/week) or with nuts (30 g/day). After a 4.1-year follow-up, participants assigned to the two MeD without calorie restriction had a 40% (significant) and 18% (non-significant) reduction, respectively, in the risk of diabetes compared with a low-fat control diet, proving the pivotal role of olive oil in the MeD [46].

A recent meta-analysis of perspective studies identified the two inflammatory markers IL-6 and C-reactive protein (CRP) as significantly associated with diabetes, with an increased risk of 26% associated with elevated CRP, and 31% due to elevated IL-6 levels [47]. The MeD has been found to be the more efficacious dietary pattern associated with significant reduction of both pro-inflammatory cytokine levels and intracellular adhesion molecule-1, decreasing inflammation and improving endothelial function [48]. The positive effect of the MeD has been shown also in the prevention of metabolic syndrome, as described in three prospective studies in which higher adherence to the MeD was associated with a reduced risk, from 15% to 18%, of developing metabolic syndrome [49,50,51].

An analysis of the Sicilian diet of centenarians from the Sicani Mountains and the eating habits of the inhabitants of Palermo showed that the low animal protein content and low glycemic index of the Sicilian MeD might directly modulate the insulin/IGF-1 and the mTOR pathways, which might be key pathways by which MeD exerts its beneficial effects in aging and longevity [52]. Specifically, the reduction of animal protein intake may significantly reduce serum IGF-1 concentrations and inhibit mTOR activity with a downregulation of the signal that leads to the FOXO3A activation and, consequently, to the transcription of homeostatic genes that favor longevity [52].

In the Nurses’ Health Study, where “healthy aging” was defined as survival to ≥70 years free of major chronic diseases, with no impairment in cognition, no physical disabilities and intact mental health, greater adherence to MeD was significantly related to better health and wellbeing in elderly subjects [53]. Moreover, two very recent meta-analyses suggested the beneficial effects of MeD on cognitive function in the elderly [54,55]. The first meta-analysis, which assessed the association between the MeD and mild cognitive impairment (MCI) or Alzheimer’s disease (AD) from five perspective cohort studies with at least one year of follow-up, points out a reduced risk (33% lower) of developing both MCI and AD, and reduced progression from MCI to AD in the case of higher MeD adherence [54]. The second meta-analysis evaluated the association between MeD and a number of brain-related conditions, such as stroke, depression, and cognitive impairment (eight studies covered cognitive impairment), suggesting that high MeD adherence was significantly associated with a reduced risk of MCI, dementia, and AD [55].

Interestingly, greater MeD adherence resulted in better scores on several cognitive function tests in elderly subjects (>60 years) living in a Polish rural community [56]. This result is important and may have significance for health policy and practice, since it shows there were benefits even in non-Mediterranean populations when the MeD was adopted. Conversely, in elderly subjects (> 60 years) enrolled in the Australian Diabetes, Obesity, and Lifestyle Study, cognitive function was found to be adversely affected by Western dietary patterns as opposed to the consumption of a vegetable- and plant-based diet [57].

Furthermore, recent studies have shown an association between telomere length, telomerase activity, and adherence to MeD in terms of its effects on the aging process and health [58]. Interestingly, there were not individual MeD components but the whole MeD that appeared to be significantly associated with leukocyte telomere length, emphasizing the role of a whole MeD pattern on health [59]. In this context, a more recent *in vitro* study showed that the MeD protects cells from oxidative stress, preventing cellular senescence and cellular apoptosis and reducing telomere attrition [60].

The rate of telomere shortening is an important biomarker of senescence; eating nutrient-rich foods might modulate telomere length and delay aging by reducing the occurrence of chronic diseases [61,62]. Nonetheless, although telomere length may be a useful clinical predictive tool, cells with shortened telomeres may remain genetically stable if the telomere maintenance system is efficiently working [63]. Thus, further data are expected to better understand the significance of the different components of the enzymatic complex involved in the telomere protective function against genome instability-promoting events. 

## 5. Sirt-Inducing Foods in the Mediterranean Diet

Different main components of the MeD may have a positive effect on longevity and healthy aging by acting on the pivotal SIRT1 signaling pathway. In particular, the beneficial effects of resveratrol, a polyphenol contained in grapes, some nuts and dried fruits, and red wine, have been related to SIRT1 activation [64,65]. Specifically, resveratrol supplementation in a mouse experimental model of AD (senescence-accelerated mouse-prone 8, SAMO8 mice) increased mean life expectancy and maximal life span in SAMP8 and in their control, the related strain senescence-accelerated mouse-resistant 1 (SAMR1), activated AMP-activated protein kinase (AMPK) pathways and pro-survival routes such as SIRT1 [66]. Neuroprotection by resveratrol requires SIRT1, with reduced acetylation of the SIRT1 substrates PGC-1alpha and p53 [67]. Some resveratrol protective effects on cancer are mediated by SIRT1 [68]. A recent experimental report demonstrated that resveratrol activates SIRT-1, which deacetylates NFkB-p65 at lysine 310 and histone 3 (H3) at the lysine 9 position, which in turn leads to decreased binding of NFkB-p65 to DNA and attenuates oxidative stress at the cardiac level through reduced transcription of NADPH oxidase subunits [69]. Other recent data showed that a low dose of resveratrol induced vascular smooth muscle cells’ (VSMC) differentiation through the stimulation of SIRT1 and AMPK. However, a low or high dose of resveratrol acts by targeting different signaling molecules implicated in the induction of VSMC differentiation; the low dose stimulates differentiation through SIRT1-mediated activation of Akt, whereas high-dose resveratrol stimulates differentiation through AMPK-mediated inhibition of the mTORC1 pathway, allowing activation of Akt [70]. Moreover, acting as a SIRT1 activator, resveratrol regulates the anti-aging effects associated with the silencing activity at telomeres, thus stabilizing DNA [71]. 

The polyphenol quercetin, contained in red wine and red onions, can also induce SIRT1; in a very recent *in vitro* study, quercetin combats oxLDL-induced endothelial oxidative damage by activating SIRT1 and modulating the AMPK/NADPH oxidase/Akt/endothelial NO synthase signaling pathway [72]. Quercetin also increases SIRT1 expression in endothelial progenitor cells (EPCs), which contribute to vascular repair after damage [73].

Moreover, polyphenols in extra-virgin olive oil are able to activate Nrf2-dependent gene expression, such as SIRT1 and increase paraoxonase (PON) activity [74,75,76].

## 6. Exercise as an Antioxidant and Sirt1 Trigger, and Its Reinforcement on Diet for a Healthy Mediterranean Lifestyle

Nutrition and physical activity represent closely interrelated basic necessities to ensure the survival of humans and animals [77]. An example of this close interaction and its effects on health may help us to understand the origin of many modern chronic diseases. In fact, when our ancestors were hunter-gatherers in the “wild” physical activity was obligatory for food supply, whereas exercise was necessary for survival. In this environment, energy was stored as glucose and glycogen to overcome the problem of the discontinuous nature of intake compared with the continuous demand. Intake was also adjusted to meet immediate changes in demand. These metabolic adaptations suggest that certain “thrifty” genotypes were selected because of their advantage over the less efficient to regulate an oscillating enzymatic control of fuel storage and use during feast/famine and physical activity/rest cycles [78]. In this context, insulin resistance during metabolic derangement was crucial, since a certain degree of insulin resistance gave advantages during periods of famine, such as increased fat oxidation for energy, maintenance of a certain “glycemic stability” and muscle glycogen conservation [79]. An efficient storage of fuel and, more important, its efficient utilization allowed for energy availability when the necessity for food required intense physical activity to hunt over great distances despite a prolonged fasted state [78]. Once the hunt was complete, feasting occurred once again, and exhausted fuel stores were restored for another cycle [78]. The onset of a society characterized by behavioral modifications (a sedentary lifestyle and an unlimited supply of food) eliminated the cycles of feast/famine and physical activity/rest [79]. This change occurred in a time period too short to allow for genomic and metabolic adaptation, thus leading to the spread of insulin resistance, obesity, Type 2 diabetes, and metabolic syndrome in our industrial societies.

To underline the close interaction between nutrition and exercise, the most recent MeD concept proposed by the UNESCO as well as by the Mediterranean Diet Foundation refers more to a “Mediterranean lifestyle” than a simple dietary pattern, where nutritional, cultural, and lifestyle factors including physical activity are considered all together as critical components of the agricultural and rural Mediterranean model [3,4]. In particular, regular physical activity (at least 30 min/day, depending on age, sex, and individual characteristics) is also recommended together with dietary advice [4].

Exercise has been identified as a modulator of oxidative stress in a pattern described by hormesis, in which the two extremes of the curve, physical inactivity or overtraining, are both potentially dangerous, whereas regular exercise induces an oxidative stress-mediated adaptation, increased resistance to oxidative stress, and enhancement of antioxidant and damage repair enzymes [32]. It has been observed that regular chronic exercise decreases oxidative stress in different categories of athletes, and that elite athletes have a longer life span when compared to the general population [80,81,82]. Nonetheless, these beneficial effects are also evident in sedentary individuals, and may be reinforced by a healthy lifestyle, which clearly includes a healthy dietary pattern. In sedentary subjects, age-dependent decline in endothelial and microvascular integrity may be reversed when combining exercise with MeD in an eight-week intervention [83,84]. In subjects with metabolic syndrome, the combination of MeD with moderate-to-high intensity training induces greater improvement in health-related quality of life with a greater effect on physical and functional fitness, body weight, enhanced EPCs, and decreased insulin sensitivity, triacylglycerols, and blood pressure than diet alone [85,86,87]. A combination of MeD, not smoking, and physical activity was associated with a lower rate of all-cause and cause-specific mortality than each factor alone [88,89].

Moreover, recent experimental data suggest that a diet rich in oleic acid, the major component of extra-virgin olive oil, can improve the adaptive response in terms of oxidative stress changes (hydroperoxides and thiobarbituric acid-reactive substances) after exhaustive exercise (forced running in a five-lane 10° inclined treadmill at a speed of 30 m/min for 70–75 min) in rats [90].

Exercise by itself is a SIRT1-activator, as shown by experimental studies [91,92]. Moreover, SIRT1 expression was increased in muscle biopsy samples of healthy untrained subjects subjected to an exercise regime (1 h, 2.4 times/week for 12 weeks; and 1.3 times/week for another 52 weeks) [93]. The anti-aging benefits of exercise appear to be mediated by the modulation of the SIRT1–AMPK pathway at cardiac and cerebral levels in animal studies [94,95].

Interestingly, in a recent study on 18-month-old rats who underwent swimming exercise training, resveratrol treatment (15 mg/kg/day), or exercise training with resveratrol treatment for one month, the PI3K–Akt pathway results increased with exercise training and resveratrol treatment, and SIRT1 was activated only with resveratrol treatment [96]. Moreover, the exercise training plus resveratrol group benefited from SIRT1 and PI3K–Akt dual pathways and blocked FOXO3 accumulation, suggesting that exercise training can reinforce resveratrol treatment’s beneficial effects in aging rat hearts [96].

## 7. Conclusions

General agreement on how healthy aging can be obtained and maintained has not yet been reached, but the MeD appears to be effective at reducing the risk of mortality and disease outcomes. Even more importantly, the concept of synergy between factors within the Mediterranean pattern encourages future studies to evaluate the impact of a comprehensive lifestyle, not only to obtain a longer life span, but—even more important—a better experience of aging, beyond the biological, psychological, and social challenges that old age entails.

**Figure 3:**
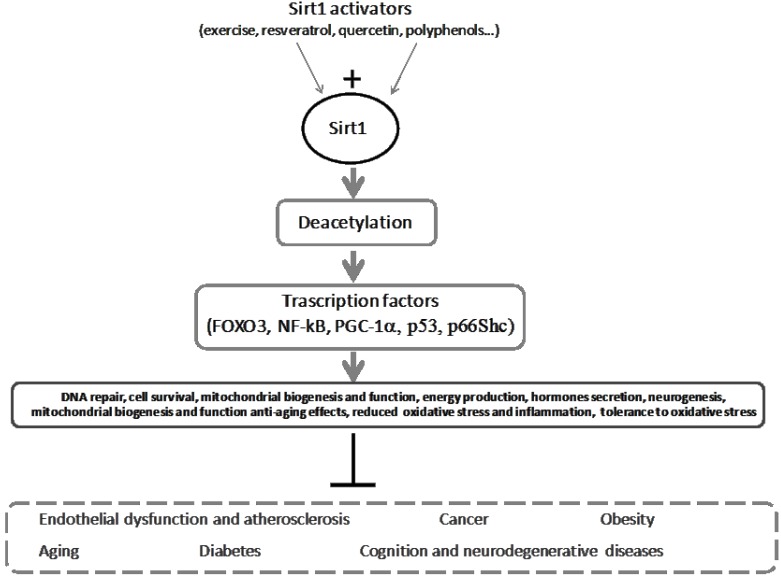
Schematic actions for SIRT1. SIRT1 modulates several different cellular functions by deacetylating transcription factors, thus reducing the onset and progression of chronic degenerative diseases and aging processes.

Of the many available oxidative biomarkers, SIRT1, which appears to be a key factor in the modulation of many vital cellular processes and oxidative stress, could represent an attractive target for SIRT1-activators (nutritional components as well as exercise) in the Mediterranean lifestyle to contrast aging and age-associated comorbidities (Figure 3).

For now, many aspects of this research area require better knowledge, and in the future efforts should be taken to validate and standardize assays to evaluate oxidative stress biomarkers, to explore the effects of the whole cluster of components in the Mediterranean lifestyle in relation to healthy aging, and to understand the mechanisms of SIRT1 activation and the connections between the SIRT genotype and disease phenotype and prognosis.
